# A cross-sectional survey study on the correlation analysis of nutritional status and intestinal flora in patients with esophageal cancer

**DOI:** 10.3389/fnut.2024.1424039

**Published:** 2024-07-12

**Authors:** Li LiYa, Zhang XinSheng, Huang Xiang, Liu Zhao, Liu Lu, Lv XiuMing, Li Ye, Chen Jing, Zhang KeMing, Wang HongChi, Xia Jing, Cong Yang, Cui Xiu, Long HongBo, You ShuQin, Liu Fang, Liu YingHua

**Affiliations:** ^1^Department of Nutrition, The First Medical Center, Chinese People's Liberation Army (PLA) General Hospital, Beijing, China; ^2^Department of Radiation Oncology, Fifth Medical Center, Chinese People's Liberation Army (PLA) General Hospital, Beijing, China; ^3^Department of Clinical Nutrition, Hebei Yanda Hospital, Langfang, China; ^4^Department of Nutrition, First Teaching Hospital of Tianjin University of Traditional Chinese Medicine, Tianjin, China; ^5^Department of Endocrinology, Jiangyin Hospital of Traditional Chinese Medicine, Wuxi, China; ^6^Department of Nutrition, The Second Affiliated Hospital of Guilin Medical University, Guilin, China; ^7^Department of Nutrition, Fangshan Hospital Beijing University of Chinese Medicine, Beijing, China; ^8^Department of Endocrinology, Ili Kazakh Autonomous Prefecture, Huocheng County Hospital of Traditional Chinese Medicine, Yili, China

**Keywords:** esophageal cancer, malnutrition, PG-SGA, NRS2002, intestinal flora

## Abstract

**Objective:**

This study aims to examine the nutritional status of individuals diagnosed with esophageal cancer and compare the nutritional indicators and intestinal flora between malnourished and non-malnourished patients. The findings aim to contribute to the early prevention of malnutrition and the development of interventions targeting the intestinal flora to treat esophageal cancer.

**Methods:**

An 80-patient sample of hospitalized individuals with esophageal cancer was selected from the radiotherapy department of our hospital between July 2021 and July 2022 to evaluate NRS2002 scores and PG-SGA scores. This cross-sectional analysis aimed to examine the disparities in dietary nutrient intake, blood indicators, body composition, and fecal intestinal flora between malnourished and non-malnourished patients with esophageal cancer. Additionally, we randomly selected 40 cases to predict and analyze the relationship between intestinal flora and malnutrition.

**Results:**

The incidence of nutritional risk and malnutrition in patients with esophageal cancer was 62.5% and 60%, respectively. The low intake of carbohydrates and dietary fiber in the malnutrition group was statistically significant compared to those in the non-malnutrition group (*P* < 0.05). The albumin (ALB) level was lower in the malnutrition group than in the non-malnutrition group, while the C-reactive protein (CRP) level was higher; these differences were also statistically significant (*P* < 0.05). The basal metabolic rate, phase angle, body cell mass, muscle mass, skeletal muscle index, and fat-free mass index in the malnutrition group all decreased compared to the non-malnutrition group. The extracellular water/total body water was higher than that in the non-malnutrition group, which was also statistically significant (*P* < 0.05). As shown by 16S rDNA sequencing of fecal intestinal flora, there was no significant difference in α and β diversity between the malnutrition and non-malnutrition groups; at the genus level, significant differences were observed for Selimonas, Clostridioides, Dielma, Lactobacillus, and [Eubacterium]_siraeum_group. However, Dielma, Sellimonas, and Clostridioides were significantly lower in the malnutrition group than in the non-malnutrition group, while Anaerococcus, Atopobium, Eubacterium_siraeum_group, and Lactobacillus were significantly higher in the malnutrition group. Correlation analysis between different genera and clinical indicators showed that Lactobacillus was positively correlated with ALB, dietary energy, intracellular water/total body water (ICW/TBW), phase angle (PA), muscle mass (MM), skeletal muscle mass (SMM), body cell mass (BCM), basal metabolic rate (BMR), appendicular skeletal muscle mass (ASMM), total body water (TBW), fat-free mass index (FFMI), skeletal muscle index (SMI), fat-free mass (FFM), Weight, body mass index (BMI) (*r* > 0, *P* < 0.05), but negatively correlated with PG-SGA score, NRS2002 score, and extracellular water/total body water (ECW/TBW) (*r* < 0*, P* < 0.05). Based on PG-SGA, there was only a low accuracy for identifying nutrient deficiency (most areas under curve (AUC) values fell within 0.5 to 0.7, or even lower), with Lachnoclostridium's AUC being 0.688 (CI = 0.518–0.858) and Lactobacillus_salivarius_g_Lactobacillus's AUC being 0.257 (CI = 0.098–0.416). A KEGG functional analysis based on 16S data indicated potential differences affecting glucose metabolism pathways and the synthesis or division of DNA, influencing the onset, development, and prognosis of esophageal cancer patients.

**Conclusion:**

Esophageal cancer patients are more likely to be malnourished. The nutritional status of these patients is closely linked to the intake of carbohydrates and fiber, albumin levels, inflammation levels, and lean body mass. Furthermore, the patient's intestinal flora composition plays a significant role in their nutritional well-being. Consequently, modulating the intestinal flora holds promise as a potential therapeutic approach for addressing malnutrition in esophageal cancer patients.

**Clinical trial registration:**

ChiCTR2100048141

## 1 Introduction

Esophageal cancer is globally recognized as a prevalent malignant tumor. According to the International Agency for Research on cancer's (IARC) statistical report in 2020 it is the eighth most common cancer, with 604,000 new cases, and the sixth leading cause of cancer-related deaths, with 544,000 deaths (1). This signifies that esophageal cancer will subsequently impose substantial economic and health burdens on China and the rest of the world over the next several decades.

Chronic esophageal cancer patients grapple with serious nutritional problems due to local tumor obstruction and destruction, systemic reactions caused by abnormal metabolism of tumor cells, and complications arising from antineoplastic therapies. It is considered to be have the highest incidence of nutritional risk, the rate of which, as reported globally, ranges between 67.5% and 78.9% (2, 3). Studies reveal that a significant proportion (60%−85%) of these patients are malnourished, the most common form of which is across all cancers (4, 5). Malnutrition in esophageal cancer impairs organ function, amplifies surgical risks, augments complications, and diminishes both short-term and long-term treatment outcomes (6). Furthermore, malnutrition reduces radiosensitivity and chemotherapy sensitivity (4, 7), decreases patient quality of life (8, 9), extends hospital stays (10), and precipitates readmission within a brief period (11). Optimal nutritional status significantly impacts the survival outcome of patients with cancer (2, 3, 12, 13), benefiting their prognosis and mitigating adverse reactions during treatment to enhance their quality of life. The European Society of Parenteral and Enteral Nutrition clearly pointed out the important role of nutritional support therapy in the comprehensive treatment of cancer patients (14). Currently, numerous national and international studies robustly validate nutrition intervention's positive influence on the nutritional status of esophageal cancer patients, its ability to decrease blood system toxicity and gastrointestinal responses, and improve therapeutic tolerance and immunity (8, 15–19). Providing nutritional support to these patients can maintain or restore their nutritional status, augment their tolerance to treatment, lessen the risk of complications, hasten recovery, and curtail hospital stays, potentially saving lives (20).

Despite optimal nutrition interventions, esophageal cancer patients may still experience poor treatment outcomes due to tumor characteristics. The Human Microbiome Project (HMP), initiated in 2007, ignited research on microbiomes. Intestinal flora is closely associated with prognosis in patients with esophageal cancer, such as promoting cancer susceptibility (21), enhanced inflammatory response, and shortened survival time (22). However, most efforts have focused on differences between esophageal cancer and healthy people, overlooking dissimilarities among malnutrition and non-malnutrition patient microbiota. This study wants to explore whether intestinal flora is beneficial to patients with esophageal cancer and then support patients with intestinal flora to improve their prognosis.

To better detect malnourished patients with esophageal cancer as soon as possible, provide reasonable nutritional support programs. This research aims to identify clinical and anthropometric indicators and gut microbiota changes in esophageal cancer patients with malnutrition for effective nutritional support.

## 2 Research objective and methodology

### 2.1 Research object

We selected esophageal cancer patients admitted to the Radiation Therapy Department of General Hospital, PLA, from July 2021 to July 2022 for our study. Written informed consent was obtained as per hospital protocol. This research had ethics approval with registration number ChiCTR2100048141 at the China Clinical Trial Registration Center. The content presented in this paper was part of the study, which intended to provide nutritional support. However, due to missing data, the study design was modified to analyze cross-sectional data from the participants who agreed to participate.

#### 2.1.1 Inclusion criteria

(1) Age > 18 years;

(2) diagnosed esophageal cancer pathologically;

(3) capacity to respond accurately to questionnaires; and

(4) well-informed about the diagnosis and willing to participate.

(5) At present, there is no drug treatment for esophageal cancer.

#### 2.1.2 Exclusion criteria

(1) Critically ill cardiac, pulmonary, liver, or kidney patients;

(2) presence of fever or infection;

(3) organs transplantation or concurrent primary tumor;

(4) cognitive deficit; and

(5) psychological/psychiatric conditions requiring immediate attention.

## 3 Data collection

### 3.1 General data

The radiation oncologist strictly selected the trial participants according to inclusion and exclusion criteria, notifying our nutritionist to evaluate them. Our department's nutritionist completed the patient evaluation within 24 h after admission. Our nutritionist provided a concise overview of the trial's purpose and executed informed consent with those willing to participate. Simultaneously, the nutritionist collected general information using anthropometric measurements and an electronic questionnaire, including name, age, height, weight, BMI, occupation, education level, pre-existing conditions, tumor stage, marital status, contact details, etc. [Weight and height are recorded rounded off to the nearest tenth of a kilogram and centimeter, respectively; BMI = weight (kg)/height (m)^2^].

### 3.2 Dietary survey

The patient's food intake (including food type and quantity) for the past 3 days is reviewed, with nutritionists accurately calculating total energy and nutrient intakes.

### 3.3 NRS2002 nutritional risk screening

This screening tool (23), endorsed by both the European Society for Parenteral and Enteral Nutrition and the Chinese Association for Parenteral and Enteral Nutrition, is administered within 24 h after admission by nurses. It captures nutritional scores, disease burden, and elderly status (1 point if aged over 70 years, zero otherwise). A maximum score of 3 indicates risk; below that, there is no risk.

### 3.4 PG-SGA nutrition evaluation (patient classification)

The PG-SGA evaluation (24) was designed for the nutritional status assessment of cancer patients, which was used to identify malnourished esophageal cancer patients in this study and was collected by nutritionists simultaneously when collecting general patient data. It consists of four parts: general condition (weight loss in the last 2 weeks, reduction in diet in the last week, gastrointestinal reactions, mobility), disease state and age (cancer, AIDS, pulmonary or cardiac cachexia, bedsore, open wound or sputum, and the age of trauma > 65 years), metabolic stress state (stress level, fever existence, duration, and hormone use per day), and physical examination (triceps skinfold thickness, grip strength, calf circumference, and ankle edema). In this study, we used a PG-SGA score of 4 as a cut-off value. Patients scoring <4 falls into the non-malnutrition group, while those scoring ≥4 forms the malnutrition group.

### 3.5 Body composition analysis

Multi-frequency BIA analysis via the portable body composition analyzer (NUTRILAB 003) considers total body water, muscle mass, fat-free mass, percentage of fat mass, and protein content. Measurements were conducted on the morning of the 2^nd^ day following patient enrollment, with instructions to fast for 2 h and abstain from water for 1 h.

### 3.6 Tumor patient quality of life scoring

Symptoms such as appetite, mental condition, sleep quality, fatigue levels, pain, family understanding, co-worker empathy, cancer self-awareness, treatment perceptions, daily activities, treatment side effects, and facial expressions are scored on a scale of 1 to 5. At present, life quality ratings range from <20 for poor, 21–30 for moderate, 31–40 for good, 41–50 for excellent, and 51–60 for great (25). Face expression scoring was subjectively conducted by a nutritionist based on the facial expression pain rating scale for patients with cancer pain.

### 3.7 Blood indicators

The fasting (8-h) venous blood samples are collected by nurses in our hospital. Complete blood cell tests utilized an ABX-MICROS-60 Automated Hematology Analyzer and its accompanying reagents, including hemoglobin (HB) (g/L), red blood count (RBC) (10^12^/L), white blood count (WBC) (10^9^/L), neutrophil (%), lymphocyte (%), monocyte, eosinophilic, platelet count (PLT) (10^9^/L), and CRP (mg/dL)-median (P25-P75). Biochemical assays were run on a HITACHI-7100 Full Auto Biochemistry Analyzer, using the corresponding kits, including alanine aminotransferase (ALT) (U/L), aspartate aminotransferase (AST) (U/L), total protein (TP) (g/L), ALB (g/L), blood urea nitrogen (BUN) (umol/L), serum creatinine (Scr) (umol/L), uric acid (UA) (umol/L), calcium (mmol/L), and phosphorus (mmol/L). The levels of immunoglobulin A (IgA) (mg/dL), immunoglobulin G (IgG) (mg/dL), and immunoglobulin M (IgM) (mg/dL) were determined according to kit instructions (Beijing Orco Biotechnology Co., Ltd., Beijing, China) by a microplate reader (Multiskan FC, ThermoFisher, Beijing, China). The superoxide dismutase (SOD) (U/mL) index level was determined with an enzyme label detector with commercial enzyme-linked immunosorbent assay kits (Nanjing, Jiangsu, China).

### 3.8 Gut microbiota analysis

Inform the patient of the precautions, and the patient retained the stool sample. Considering that patients with esophageal cancer have limited eating and may not defecate in the morning, the sample is selected for the patient's feces before treatment, not limited to time. Provided the patient with our department's contact information. Once the specimen is retained, our nutritionist will collect it. The specific precautions for fecal specimen collection are as follows: Scoop a pea-sized stool and place it into the tube containing the stool preservation solution. To avoid contamination, the scoop could not touch any extraneous area, including urine or other body fluids. The microbiological diversity of these samples was analyzed by Fanxing Boao (Beijing) Technology Co., Ltd. using the 16S method.

Total genomic DNA was extracted using a DNA extraction kit following the manufacturer's instructions. The concentration of DNA was verified with NanoDrop and an agarose gel. The genome DNA was used as a template for PCR amplification with the barcoded primers and Tks Gflex DNA Polymerase (Takara). Amplicon quality was visualized using gel electrophoresis, purified with AMPure XP beads (Agencourt), and amplified for another round of PCR. After being purified with the AMPure XP beads again, the final amplicon was quantified using a Qubit dsDNA assay kit. Equal amounts of purified amplicon were pooled for subsequent sequencing.

Alpha diversity analysis includes the ACE index, the Chao1 index, the Shannon index, and the Simpson index. The former two represent the richness of intestinal flora, and the latter two represent the diversity of intestinal flora. The higher the value, the higher the species richness or diversity.

Beta diversity analysis is a comparative analysis of the composition of the two groups of samples. In this study, PCoA map analysis was used to compare the differences in the composition of intestinal flora between malnutrition and non-malnutrition groups. PCoA analysis provides results based on multiple distance matrices. Through PCoA, the differences between individuals or groups can be observed. Each point in the figure represents a sample, and the same color is the same group. The closer the sample distance of the same group is, and there is a significant distance from other groups, indicating that the grouping effect is good.

Linear discriminant analysis effect size (LEfSe) analysis can show the species with significant differences in abundance in different groups and is used to analyze the effect of each species' abundance on the difference effect. In this study, species with an impact value >4 were set as biomarkers.

Correlation analysis between genus-level differential bacteria and clinical indicators.

ROC curve analysis of differential bacteria based on PG-SGA malnutrition genus level.

Further, we analyzed the effect of classification differences between malnutrition and non-malnutrition groups on function. The PICRUSt program was used to predict the differentially expressed functions and metabolic pathways between the two groups based on the KEGG function of 16S.

## 4 Statistical analysis

Excel was used to establish a database and double-entry survey data. SPSS 26.0 was used for data analysis. Statistical software G ^*^ Power (developed by the University of Düsseldorf, Germany, specifically for the calculation of statistical power and sample size statistics) was used to estimate the sample size required 34 cases, effect size = 0.5, α = 0.05, and power (1-β) = 0.9. Count data were described by frequency and percentage; the measurement data conforming to normality were described by mean ± standard deviation, and the measurement data not conforming to normality were expressed by median (25%, 75%). Frequencies were compared using the χ2 test. Means and SD values were compared with the Student's *t*-test. Correlation analysis was performed using Spearman. ROC analysis used sensitivity as the ordinate and (1-specificity) as the abscissa to construct a curve, and the cut-off point with the largest Youden index was used to determine the critical value of malnutrition. *P* < 0.05 indicated that the difference was statistically significant.

## 5 Experimental result

### 5.1 Demographic profile of 80 esophageal cancer patients

As shown in Table 1, a total of 80 patients, comprising 16 F and 64 M with an average age of 62.1 years, were included in the study. This study suggests that the average BMI of patients is at a normal level. Eighty percentage had a family history of cancer, and 87.5% presented as Stage IV esophageal carcinoma. A staggering total of 62.5% had nutritional risks, and 60% suffered from malnutrition, a majority share overall. The mean quality of life score was categorized as great.

**Table 1:** Demographic and clinical characteristics of patients with esophageal cancer (*n* = 80).

**Features**	**Esophageal cancer**
Sex (male/female)	64/16
Age (years)	62.10 ± 7.56
Weight (kg)	66.10 ± 12.12
Height (cm)	167.43 ± 8.47
Body mass index (kg/m^2^)	23.47 ± 3.19
**Educational status**	
Illiterate	6 (7.5%)
Primary school	10 (12.5%)
Middle school	46 (57.5%)
University and above	18 (22.5%)
**Socioeconomic status (annual income, RMB)**	
<¥ 50,000	18(22.5%)
¥ 50,000–¥ 100,000	40(50%)
¥ 100,000–¥ 150,000	16(20%)
¥ 150,000 +	6(7.5%)
**Drinking status**	
No	22 (27.5%)
Occasionally	10 (12.5%)
Often	10 (12.5%)
Alcoholism	38 (47.5%)
**Smoking status**	
Don't smoke or quit	44 (55%)
Quit smoking < 12 months	26 (32.5%)
Smoking	10 (12.5%)
**Exercise**	
No	30 (37.5%)
Occasionally	30 (37.5%)
Often	20 (25%)
History of diabetes	8 (10%)
History of hypertension	38 (47.5%)
History of CHD	4 (5%)
Family history of cancer	64 (80%)
**Cancer staging**	
I	6 (7.5%)
II	2 (2.5%)
III	2 (2.5%)
IV	70 (87.5%)
NRS2002 score	2.80 ± 1.40
<3	30 (37.5%)
≥3	50 (62.5%)
PG-SGA score	4.75 ± 3.16
<4	32 (40%)
≥4	48 (60%)
QOL score	53.53 ± 6.26

CHD, coronary heart disease. Exercise: No: no active activity; Often: at least 5 days of moderate-intensity physical activity per week, for more than 150 min; Occasionally: between no and often.

### 5.2 Demographic profile of 80 esophageal cancer patients classified by PG-SGA

As shown in Table 2, the NRS2002 score was significantly higher in the malnutrition group than in the non-malnutrition group (*P* < 0.05), and the QOL score was significantly lower in the malnutrition group than in the non-malnutrition group (*P* < 0.05). The malnutrition group also reported drinking significantly more than the non-malnutrition group (*P* < 0.05).

**Table 2:** Demographic and clinical characteristics of patients with esophageal cancer between the non-malnutrition and malnutrition groups (*n* = 80).

**Features**	**Total (n = 80)**	**PG-SGA**		***P***
		**Non-malnutrition group (**<**4)** ***n** =* **32**	**Malnutrition group(**≥**4)** ***n** =* **48**	
Age (y)	62.10 ± 7.56	59.38 ± 6.90	63.92 ± 7.58	0.062
Height (cm)	167.43 ±8.47	169.12 ± 8.23	166.29 ± 8.61	0.306
Weight (kg)	66.10 ±12.12	69.88 ± 10.78	63.58 ± 12.52	0.109
BMI (kg/m^2^)	23.47 ± 3.19	24.30 ± 2.17	22.91 ± 3.66	0.142
NRS2002 score	2.80 ± 1.40	1.69 ± 1.20	3.54 ± 0.98	<0.001
QOL score	53.52 ± 6.26	57.81 ± 2.20	50.67 ± 6.48	<0.001
History of diabetes	8 (10.0%)	0 (0.0%)	8 (100.0%)	0.237
History of hypertension	4 (5.0%)	2 (50.0%)	2 (50.0%)	1.000
History of CHD	38 (47.5%)	10 (26.3%)	28 (73.7%)	0.093
Family history of cancer	64 (80.0%)	28 (43.75%)	36 (56.25%)	0.572
**Sex**				0.572
Male	64 (80.0%)	28 (43.8%)	36 (56.2%)	
Female	16 (20.0%)	4 (25.0%)	12 (75.0%)	
**Educational status**				0.238
Illiterate	6 (7.5%)	2 (33.3%)	4 (66.7%)	
Primary school	10 (12.5%)	0 (0.0%)	10 (100.0%)	
Middle school	46 (57.5%)	20 (43.5%)	26 (56.5%)	
University and above	18 (22.5%)	10 (55.6%)	8 (44.4%)	
**Socioeconomic status** **(annual income, RMB)**				0.051
<¥ 50,000	18 (22.5%)	12 (66.7%)	6 (33.3%)	
¥ 50,000–¥ 100,000	40 (50.0%)	8 (20.0%)	32 (80.0%)	
¥ 100,000–¥ 150,000	16 (20.0%)	8 (50.0%)	8 (50.0%)	
¥ 150,000+	6 (7.5%)	4 (66.7%)	2 (33.3%)	
**Drinking status**				0.039
No	22 (27.5%)	4 (18.2%)	18 (81.8%)	
Occasionally	14 (17.5%)	12 (85.7%)	2 (14.3%)	
Often	6 (7.5%)	2 (33.3%)	4 (66.7%)	
Alcoholism	38 (47.5%)	14 (36.8%)	24 (61.2%)	
**Smoking status**				0.481
Don't smoke or quit	44 (55.0%)	14 (31.8%)	30 (68.2%)	
Quit smoking < 12 months	26 (32.5%)	14 (53.8%)	12 (46.2%)	
Smoking	10 (12.5%)	4 (40.0%)	6 (60.0%)	
**Exercise**				0.108
No	30 (37.5%)	14 (46.7%)	16 (53.3%)	
Occasionally	30 (37.5%)	6 (20.0%)	24 (80.0%)	
Often	20 (25.0%)	12 (60.0%)	8 (40.0%)	
**Cancer staging**				0.513
I	6 (7.5%)	2 (33.3%)	4 (66.7%)	
II	2 (2.5%)	2 (100.0%)	0 (0.0%)	
III	2 (2.5%)	2 (100.0%)	0 (0.0%)	
IV	70 (87.5%)	26 (37.1%)	44 (62.9%)	

### 5.3 Based on PG-SGA, the dietary intake and blood biochemical indexes of the malnutrition group and the non-malnutrition group were evaluated

As shown in Table 3, the intake of carbohydrates and dietary fiber was significantly lower in the malnutrition group than in the non-malnutrition group (*P* < 0.05). The inflammatory index CRP in the non-malnutrition group was significantly lower than that in the malnutrition group, and the ALB level was significantly higher (*P* < 0.05).

**Table 3:** Comparison of nutrient intake and blood biochemical indexes between non-malnutrition and malnutrition groups (*n* = 80).

**Indicators**		**PG-SGA**		***P***
		**Non-malnutrition group (**<**4)** ***n*** = **32**	**Malnutrition group (**≥**4)** ***n*** = **48**	
**Nutrient intake**				
	Energy (Kcal/d)	2,170.93 ± 349.77	1,964.92 ± 339.72	0.071
	Protein (g/d)	88.22 ± 22.04	96.31 ± 16.26	0.189
	Fat (g/d)	73.97 ± 15.51	73.32 ± 14.97	0.896
	Carbohydrates (g/d)	279.07 ± 75.66	221.45 ± 50.00	0.006
	Fiber (g/d)	12.64 ± 6.19	9.01 ± 3.72	0.026
**Blood biochemical indexes**				
	HB (g/L)	129.81 ± 15.13	124.42 ± 24.52	0.438
	RBC (10^12^/L)	4.23 ± 0.48	4.07 ± 0.63	0.416
	WBC (10^9^/L)	5.77 ± 1.99	6.12 ± 1.51	0.539
	Neutrophil (%)	64.96 ± 10.02	63.11 ± 10.39	0.580
	Lymphocyte (%)	24.49 ± 7.48	26.56 ± 9.30	0.462
	Monocyte	6.98 ± 2.67	7.80 ± 2.52	0.325
	Eosinophilic	3.19 ± 5.01	2.05 ± 2.16	0.330
	PLT (10^9^/L)	222.25 ± 76.88	243.25 ± 76.88	0.407
	CRP (mg/dL)-median (P_25_-P_75_)	0.25 (0.10–0.50)	0.88 (0.22–1.60)	0.033
	ALT (U/L)	19.44 ± 11.82	17.31 ± 8.63	0.513
	AST (U/L)	20.71 ± 12.78	17.33 ± 5.18	0.250
	TP (g/L)	68.62 ± 3.89	68.62 ± 4.89	0.673
	ALB (g/L)	40.74 ± 2.51	37.94 ± 2.58	0.002
	BUN (umol/L)	4.58 ± 1.66	5.13 ± 1.30	0.244
	Scr (umol/L)	72.86 ± 12.89	77.47 ± 22.94	0.471
	UA (umol/L)	340.84 ± 91.88	321.10 ± 96.98	0.524
	Calcium (mmol/L)	2.26 ± 0.09	2.30 ± 0.13	0.224
	Phosphorus (mmol/L)	1.11 ± 0.17	1.13 ± 0.21	0.836
	IgA (mg/dL)	261.81 ± 97.94	258.88 ± 92.08	0.924
	IgG (mg/dL)	1,098.25 ± 197.24	1,111.33 ± 331.86	0.888
	IgM (mg/dL)	62.06 ± 23.43	65.09 ± 28.07	0.723
	SOD (U/mL)	141.92 ± 13.72	137.15 ± 16.85	0.353

HB, Hemoglobin; RBC, Red blood count; WBC, White blood count; PLT, Platelet count; CRP, C-reactive protein; ALT, Alanine aminotransferase; AST, Aspartate aminotransferase; TP, Total protein; ALB, Albumin; BUN, Blood urea nitrogen; Scr, Serum creatinine; UA, Uric acid; Ca, Calcium; P, Phosphorus; IgA, Immunoglobulin A; IgG, Immunoglobulin G; IgM, Immunoglobulin M; SOD, Superoxide dismutase.

### 5.4 Body composition variations in the malnutrition group and the non-malnutrition group

As shown in Table 4, the basal metabolic rate, phase angle, total body water, intracellular water/total body water, total body cell mass, muscle mass, skeletal muscle index, limb skeletal muscle mass, and fat-free body mass index of patients with good nutrition were significantly higher than those in the malnutrition group (*P* < 0.05). ECW/TBW (%) extracellular water/total body water was significantly lower in the non-malnutrition group than in the malnourished group (*P* < 0.05).

**Table 4:** Comparison of body composition analysis between non-malnutrition and malnutrition groups (*n* = 80).

**Indicators**	**PG-SGA**		***P***
	**Non-malnutrition group (**<**4)** ***n** =* **32**	**Malnutrition group(**≥**4)** ***n** =* **48**	
Height (cm)	169.44 ± 8.64	166.38 ± 8.59	0.277
Weight (kg)	69.38 ± 10.74	63.88 ± 12.05	0.148
BMI (kg/m^2^)	23.97 ± 1.92	22.81 ± 3.30	0.214
BMR (kcal/d)	1,806.18 ± 320.73	1,592.93 ± 174.20	0.010
PA (°)	7.18 ± 2.07	5.76 ± 0.68	0.003
FFM (kg)	62.09 ± 12.10	55.28 ± 9.23	0.051
FFM/BW (%)	89.15 ± 7.24	87.19 ± 7.89	0.430
TBW (kg)	46.07 ± 8.79	41.08 ± 6.51	0.046
TBW/BW (%)	66.36 ± 7.50	65.01 ± 7.33	0.575
ECW (kg)	18.93 ± 2.63	19.25 ± 2.73	0.719
ECW/TBW (%)	42.00 ± 6.82	47.10 ± 3.53	0.004
ICW/TBW (%)	58.00 ± 6.82	52.90 ± 3.53	0.004
BCM (kg)	36.43 ± 11.06	29.06 ± 6.01	0.010
FM (kg)	7.29 ± 4.70	8.60 ± 5.69	0.451
FM/BW (%)	10.85 ± 7.24	12.81 ± 7.89	0.430
MM (kg)	32.90 ± 8.01	28.45 ± 5.47	0.043
MM/BW (%)	47.23 ± 8.69	45.05 ± 7.94	0.417
SMI	11.39 ± 2.14	10.10 ± 1.43	0.028
SMM (kg)	32.90 ± 8.01	28.45 ± 5.47	0.043
ASMM (kg)	24.58 ± 6.12	20.60 ± 3.75	0.015
FMI	2.54 ± 1.78	3.08 ± 2.10	0.406
FFMI	21.43 ± 2.54	19.73 ± 2.25	0.033

BMI, Body mass index; BMR, Basal metabolic rate; PA, Phase angle; FFM, Fat free mass; BW, Body mass; TBW, Total body water; ECW, Extracellular water; ICW, Intracellular water; BCM, Body cell mass; FM, Fat mass; MM, Muscle mass; SMI, Skeletal muscle index; SMM, Skeletal muscle mass; ASMM, Appendicular skeletal muscle mass; FMI, Fat mass index; FFMI, Fat-free mass index.

### 5.5 Analysis of intestinal flora diversity in the malnutrition group and non-malnutrition group

**Figure 1:**
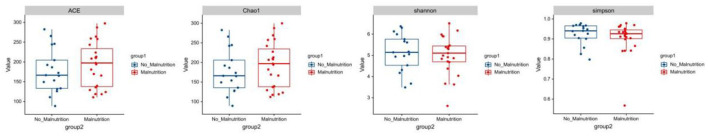
Analysis of α diversity of intestinal flora in two groups.

As shown in Figure 1, there was no significant difference in the Alpha diversity ACE index, Chao1 index, Shannon index, or Simpson index of intestinal flora between the two groups (*P* < 0.05), but there were slight differences in the richness and diversity of the two groups.

**Figure 2:**
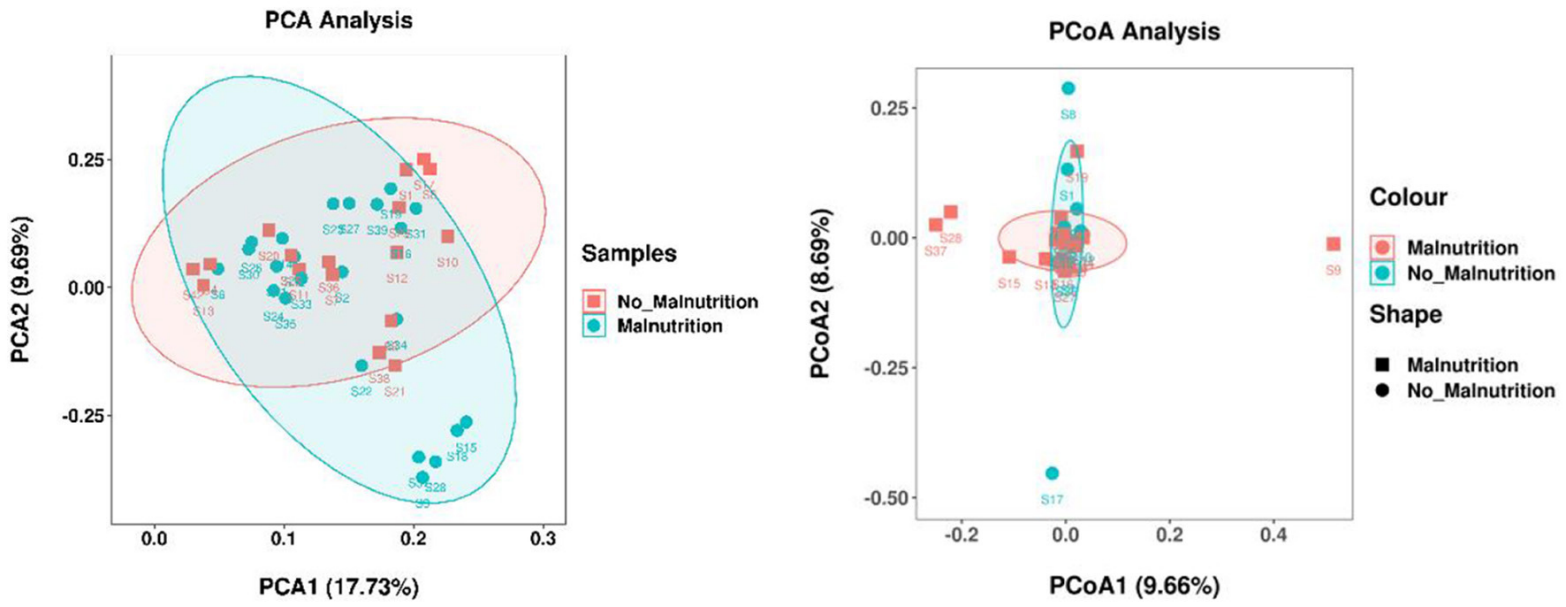
Analysis of β diversity of intestinal flora in two groups. The percentage in the figure is the interpretation rate of the principal component to the difference between the samples; a single sample is represented by a point, and the color of the same group is the same.

According to the results of PCoA analysis, as shown in Figure 2, the two groups of samples were relatively clustered, but some of the flora between the two groups was also relatively discrete, indicating that some of the flora of the two groups of samples were significantly different.

#### 5.5.1 LEfSe analysis results of two groups at the genus level

**Figure 3:**
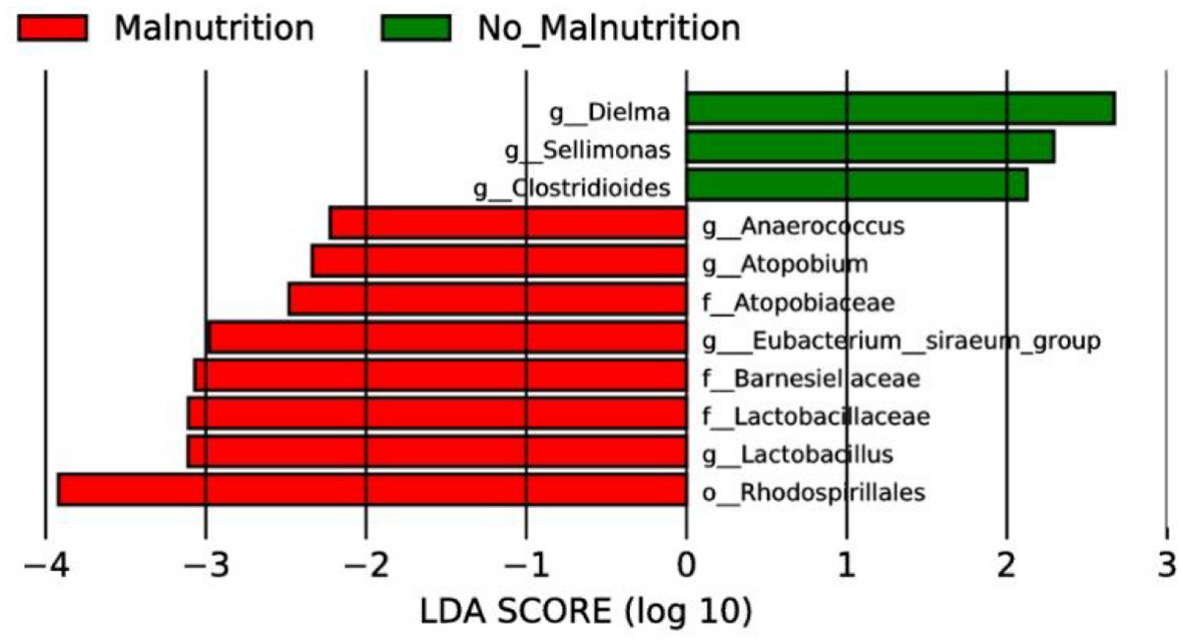
LEfSe analysis of two groups. In this study, species with an impact value greater than 4 were considered biomarkers.

As shown in the following Figure 3, the results of LEfSe analysis showed that at the genus level, the genera with significantly increased relative abundance in the non-malnutrition group were Dielma, Sellimonas, and Clostridioides, respectively. The genera with significantly reduced relative abundance were Anaerococcus, Atopobium, Eubacterium _ siraeum _ group, and Lactobacillus, respectively.

#### 5.5.2 Correlation analysis between genus-level differential bacteria and clinical indicators

**Figure 4:**
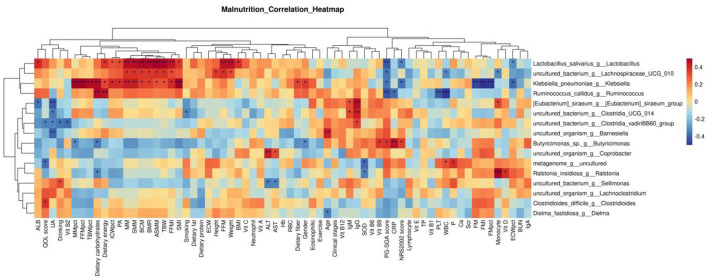
Correlation analysis between differential bacteria at the genus level and clinical indicators. *p* ≥ 0.05 unmarked; ^*^0.01<*p*<0.05; ^**^0.001<*p*<0.01; ^***^p≤0.001.

As shown in the following Figure 4. The results showed that Sellimonas was positively correlated with Drinking (*r* = 0.322, *P* = 0.042) and negatively correlated with ALT (*r* = –0.346, *P* = 0.029) and AST (*r* =−0.333 0, *P* = 0.036). The genus Clostridioides was positively correlated with the QOL score (*r* = 0.377, *P* = 0.016). The genus Dielma was negatively correlated with Age (*r* = –0.346, *P* = 0.026). Lactobacillus was positively correlated with ALB (*r* = 0.394, *P* = 0.012), Dietary energy (*r* = 0.370, *P* = 0.019), ICWpct (*r* = 0.315, *P* = 0.048), PA (*r* = 0.321, *P* = 0.043), MM (*r* = 0.469, *P* = 0.002), SMM (*r* = 0.469, *P* = 0.002), BCM (*r* = 0.484, *P* = 0.002), ASMM (*r* = 0.513, *P* = 0.001), TBW (*r* = 0.488, *P* = 0.001), FFMI (*r* = 0.446, *P* = 0.004), SMI (*r* = 0.367, *P* = 0.020), Weight (*r* = 0.456, *P* = 0.003), and BMI (*r* = 0.388, *P* = 0.013). It was negatively correlated with PG-SGA score (*r* = –0.424, *P* = 0.006), NRS2002 score (*r* = –0.334, *P* = 0.035), and ECWpct (*r* = –0.315, *P* = 0.048). [Eubacterium] _ siraeum _ group was positively correlated with IgM (*r* = 0.326, *P* = 0.040), IgG (*r* = 0.467, *P* = 0.002), and monocyte (*r* = 0.332, *P* = 0.036), but negatively correlated with ALB (*r* = –0.370, *P* = 0.019) and UA (*r* = –0.419, *P* = 0.007).

#### 5.5.3 ROC curve analysis of differential bacteria based on PG-SGA malnutrition genus level

**Figure 5:**
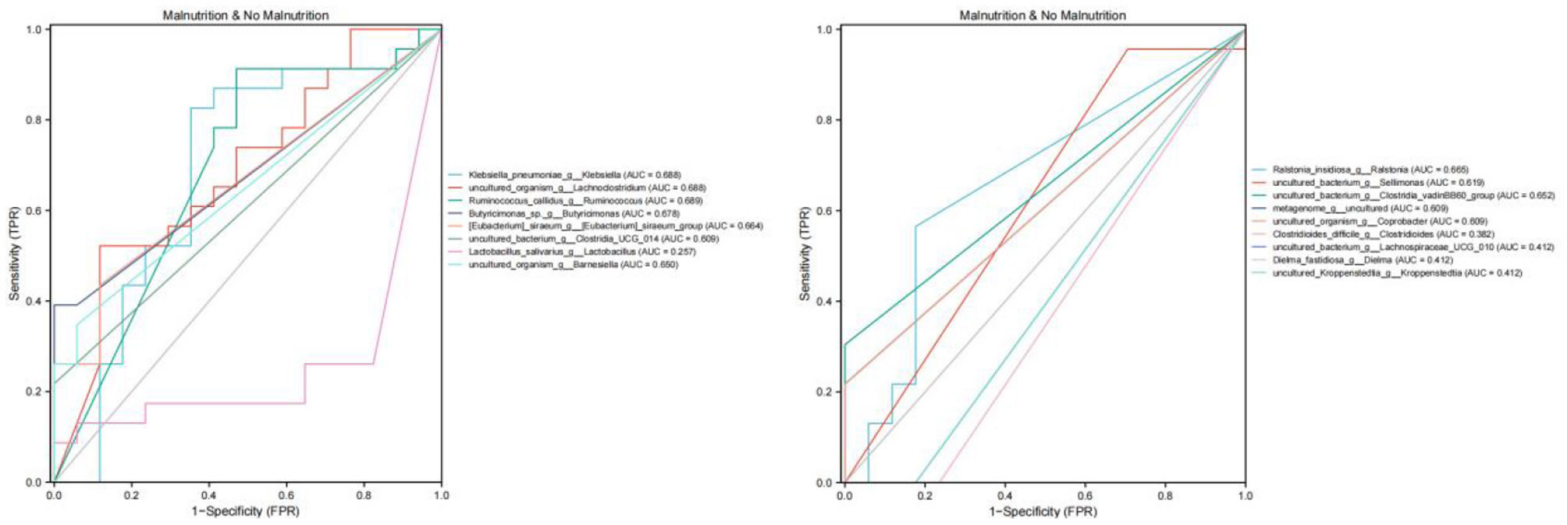
ROC curve analysis based on PG-SGA malnourished genus level differential bacteria. The area under the ROC curve is between 0.5 and 1. The closer the AUC is to 1, the better the diagnostic effect is. AUC has low accuracy when 0.5 ~ 0.7, AUC has certain accuracy when 0.7 ~ 0.9, and AUC has high accuracy when above 0.9.

As shown in Table 5 and Figure 5, the efficacy of [Eubacterium] _ siraeum _ group, Lactobacillus, Barnesiella, and Sellimonas genus levels in identifying malnutrition in patients with esophageal cancer only has low accuracy.

**Table 5:** ROC curve was used to analyze the efficacy of differential bacteria at the genus level in identifying malnutrition in patients with esophageal cancer.

**Bacteria**	**AUC**	**95% CI**
Klebsiella_pneumoniae_g__Klebsiella	0.688	0.504–0.872
uncultured_organism_g__Lachnoclostridium	0.688	0.518–0.858
Ruminococcus_callidus_g__Ruminococcus	0.689	0.531–0.848
Butyricimonas_sp._g__Butyricimonas	0.678	0.564–0.791
[Eubacterium]_siraeum_g__[Eubacterium]_siraeum_group	0.664	0.535–0.793
uncultured_bacterium_g__Clostridia_UCG_014	0.609	0.523–0.695
Lactobacillus_salivarius_g__Lactobacillus	0.257	0.098–0.416
uncultured_organism_g__Barnesiella	0.650	0.536–0.763
Ralstonia_insidiosa_g__Ralstonia	0.665	0.508–0.822
uncultured_bacterium_g__Sellimonas	0.619	0.499–0.739
uncultured_bacterium_g__Clostridia_vadinBB60_group	0.652	0.556–0.748
metagenome_g__uncultured	0.609	0.523–0.695
uncultured_organism_g__Coprobacter	0.609	0.523–0.695
Clostridioides_difficile_g__Clostridioides	0.382	0.278–0.486
uncultured_bacterium_g__Lachnospiraceae_UCG_010	0.412	0.318–0.505
Dielma_fastidiosa_g__Dielma	0.412	0.318–0.505
uncultured_Kroppenstedtia_g__Kroppenstedtia	0.412	0.318–0.505

AUC, area under the curve.

#### 5.5.4 Function prediction of malnutrition group and non-malnutrition group

**Figure 6:**
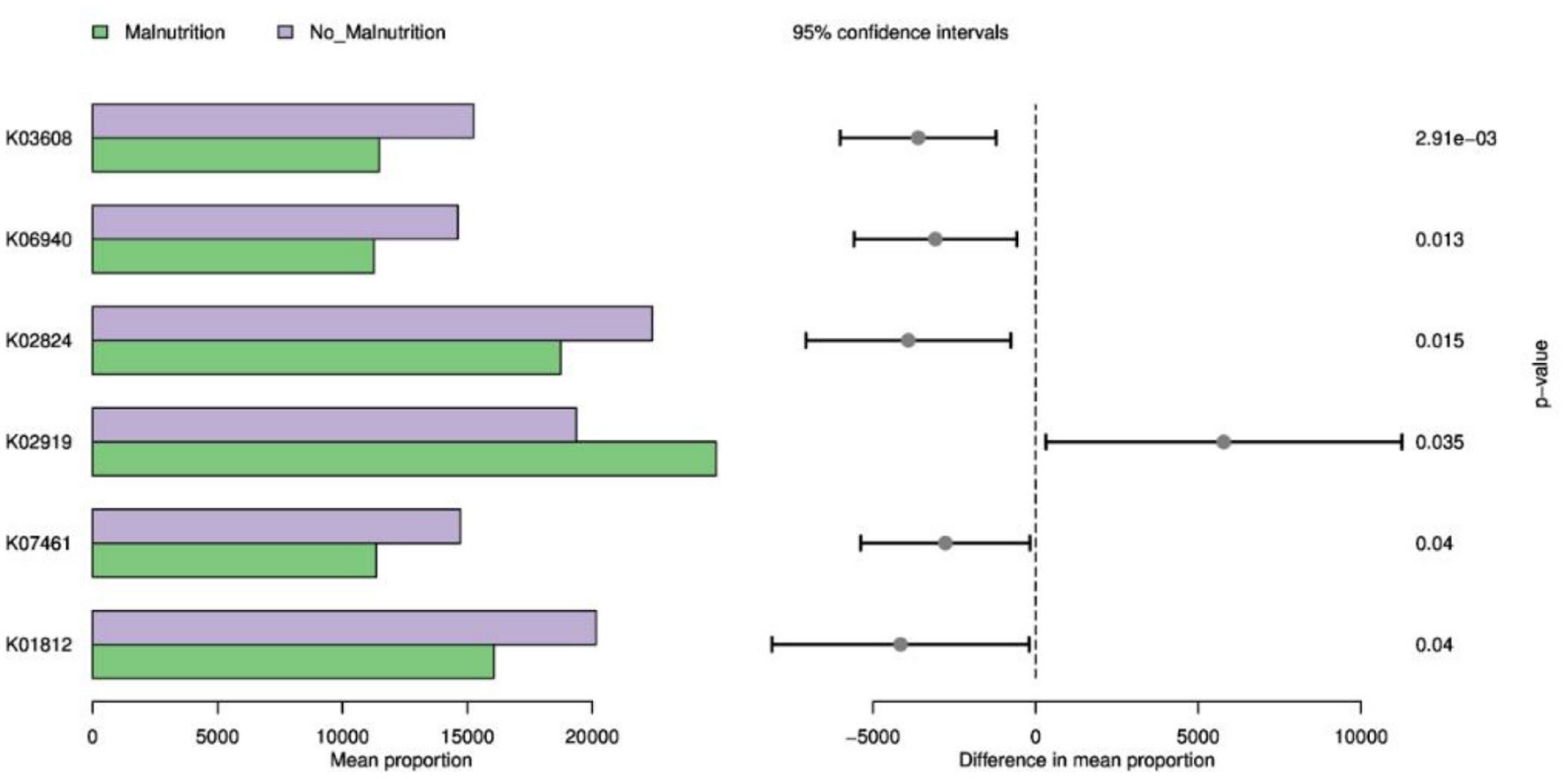
KEGG function prediction based on 16S. K03608: minE; cell division topological specificity factor; K06940: K06940; uncharacterized protein; K02824: pyrP, uraA; uracil permease; K02919: RP-L36, MRPL36, rpmJ; large subunit ribosomal protein L36; K07461: K07461; putative endonuclease; K01812: uxaC; glucuronate isomerase [EC:5.3.1.12].

As shown in Figure 6, Cell division topological specificity factor, K06940; uncharacterized protein, pyrP, uraA; uracil permease, K07461; putative endonuclease, uxaC; glucuronate isomerase [EC: 5.3.1.12] pathway expression decreased, while RP-L36, MRPL36, rpmJ, and the expression of the large subunit ribosomal protein L36 pathway were increased.

## 6 Discussion

Esophageal cancer patients demonstrate high incidences of both nutritional risk and malnutrition, particularly among older people. Clinical screening should be performed promptly with these indicators as a basis for parenteral nutrition intervention (26). This study was a cross-sectional study. All patients were fed orally at the time of enrollment. Our research identified elevated nutritional risks and malnutrition prevalence in esophageal cancer hospitalized individuals; lower carbohydrate and dietary fiber intake was also noted in malnourished patients. Findings implicated nutrition deficiency facilitating low albumin or lean body weight, elevated inflammation markers, and a specific gut microbiota profile negatively correlated with PG-SGA and NRS2002 scores yet positively associated with ALB, PA, FFMI, SMI, FFM, weight, and BMI. Based on PG-SGA, there was only a low accuracy for identifying nutrient deficiency (most AUC values fell within 0.5 to 0.7, or even lower). Further exploration is warranted based on an expanded sample size to assess potential progressivity. We also uncovered that esophageal cancer patients' malnutrition impacts glucose metabolism and aspects of DNA synthesis/division metabolic pathways; these findings contribute to the understanding of esophageal cancer patients' malnutrition diagnosis, nutritional intervention, and clinical application of gut microbiome analysis.

The present study included patients with an 80% family history of cancer, most notably 87.5% with stage IV esophageal cancer, aligning well with the observed characteristics of esophageal cancer; that is, once found, they may be patients with advanced stage. Moreover, 62.5 % have nutritional risk, accounting for more than half. Concurrently, there was a significant prevalence of malnutrition at 60%, which constitutes more than half of the population. The QOL score was significantly lower in the malnutrition group. Drinking may be a risk factor for malnutrition in patients with esophageal cancer.

Tumorigenesis intertwines with environmental factors, of which dietary and lifestyle attributes rank as the most critical (27, 28). This study revealed that the non-malnutrition group consumed more carbohydrates and dietary fiber than the malnutrition group, conveying significance. However, other nutritional aspects were comparable. The results of this study showed that high carbohydrate intake might play an important role in the nutritional status of patients with esophageal cancer. However, so far, the range of carbohydrate intake and the role of carbohydrate quality (29, 30) in the occurrence and development of esophageal cancer still need to be further explored. Dietary fiber, derived from vegetables, fruits, grains, and soybeans, is indigestible by the human small intestine (31, 32) possessing anticancer properties (33, 34). In addition, some studies have shown that high-fiber diets can improve metabolic functions within the gut microbiome and suppress carcinogen production, thereby reducing the risk of esophageal cancer. This study showed that dietary fiber intake was negatively correlated with the occurrence of malnutrition in patients with esophageal cancer. Although dietary fiber intake did not reach the daily recommended intake of Chinese residents, statistical differences were still consistent with the results of related studies (34–36). Collectively, a high-fiber diet may provide a protective shield against esophageal cancer and protect against the occurrence of malnutrition in esophageal cancer patients.

Our discoveries showed that esophageal cancer patients without malnutrition had lower levels of the inflammatory marker CRP and higher levels of ALB than those with malnutrition. In recent years, the correlation between high CRP levels and malignancies has garnered significant attention. Numerous cancers display higher CRP, reflecting both systemic inflammatory response and tumor progression, both significantly linked to patient prognosis and survival (37). The 2018 GLIM standard also uses inflammation indicators (including CRP) as the etiological criteria for the diagnosis of malnutrition. This study also confirmed malnutrition in patients with high CRP. Therefore, for patients with esophageal cancer with high CRP, whether nutritional support can be given in advance to improve the prognosis and survival of patients remains to be further explored. At the same time, most of the patients with esophageal cancer are the elderly, who experience a significant decline in vital organ functions due to multi-morbidity, extensive disease duration, elevated consumption, surgical trauma, and inadequate nutrient intake. The condition commonly results in negative nitrogen balance and low serum albumin levels (38). Also, it's worth noting that preoperative albumin levels have been found to correlate with patient outcomes. That is, patients with lower albumin levels tend to have less favorable prognoses (39–41), leading to increased postsurgical respiratory complications, wound infections, or anastomosis fistulae rates compared to those with normal albumin levels (42).

Furthermore, these patients experience longer hospital stays post-surgery and more frequent relapses, resulting in unfavorable outcomes and secondary surgeries (43). The possible mechanism is as follows: Hypoalbuminemia impairs host immunity, while muscle wasting and fat consumption can result in respiratory muscle weakness, undermining ventilation and gas exchange function; protein is crucial for wound healing. Insufficient protein intake delays wound healing and can contribute to local tissue edema, hindering wound repair and facilitating infection. Hence, monitoring albumin levels and implementing nutritional support can significantly reduce postoperative complications, simplify hospital and surveillance periods, and improve the prognosis. In summary, serum CRP and albumin levels act as reliable biomarkers, while high serum CRP and hypoalbuminemia are independent prognostic factors in esophageal cancer and can predict the survival of patients with esophageal cancer to a certain extent.

Body composition analysis is a scale to assess human components and functionality. It aids in diagnosing the nutritional state of cancer patients, monitoring their dynamics, evaluating interventions, and improving their quality of life. Emerging research (44, 45) indicates marked compositional changes in cancer patients throughout their illness. Numerous studies endorse an association between body composition and survival outcomes and a better understanding of how body composition is used to evaluate the prognosis of cancer patients. Our study found that non-malnourished patients displayed higher base metabolic rates (BMR), phase angles, muscle mass, skeletal myofiber index, limb lean tissue mass, and fat-free weight index than malnourished ones; these differences were statistically significant, *P* < 0.05, echoing previous findings (46–48). Recently, it was discovered that poor nutrition in cancer often manifests as severe muscle mass (MM) depletion at any stage, predicting poor physical function, lower quality of life, surgical complications, disease progression, and survival rate (46, 49–52). A high prevalence of low MM is observed in new cancer cases >50%, significantly surpassing healthy individuals around this age by approximately 65% (53). Reversing low MM could improve cancer treatment outcomes, morbidity, and, ultimately, mortality rate (53). Given the role of MM tissue in oncological outcomes, strategies to optimize body composition are an important part of successful cancer treatment, and nutrition is one such way to beneficially influence MM tissue. This can, in turn, improve general health and outcomes, including treatment and tolerance for survival (46, 47). The phase angle (PA) (54) is a highly sensitive marker for detecting patient malnutrition and predicting the outcomes of various diseases. It encapsulates bodily tissue attributes related to diseases, nutrition statuses, and hydration levels, allowing a holistic assessment of health and nutritional status. Compared with conventional nutritional evaluation tools, PA has unique advantages in nutritional assessment, efficacy monitoring, and prognosis prediction for patients with malignant tumors. It has broad application prospects in clinical practice. PA provides rapid measurements within 3 min, making it applicable even for individuals with abnormal shapes (54). Numerous studies show that (55–58) PA decreases with the aggravation of malnutrition. The larger the PA, the more complete the cell membrane is and the stronger the cell functions. Low levels of PA have been identified as poor prognostic factors affecting survival in diverse types of cancer patients across multiple body sites. Research indicates that lower PA correlates with elongated hospital stays, a significant decrease in survival time, a higher incidence of postoperative complications, and heightened mortality risks among tumor patients (55). The probable mechanism lies largely in the ECW/TBW ratio. Adjusting PA by this ratio could enhance prognosis and eventually improve palliative care in cancer cachexia patients, necessitating the determination of an optimal cut-off value for PA detection of malnutrition among cancer patients. Currently, minimal literature exists about the influence of body composition parameters, such as BMI, body fat percentage, SMI, and sarcopenia, on postsurgical morbidity and long-term survival in esophageal carcinoma patients. This study furnishes preliminary data on human body component parameter changes in esophageal carcinoma patients, facilitating prospective and extensive clinical trials based on body component analysis results for nutritional supplement dose standardization among cancer patients, thus achieving “precision” nutritional intervention.

Gut microbiota research has surged since the advent of next-generation sequencing, illuminating its role in health and disease. Evidence links it to various cancers (59, 60), potentially offering novel cancer therapies targeting the gut microbiome. However, research on esophageal cancer related to gut microbiota is primarily cross-sectional between patients and controls (61). Specifically, there remains a dearth of data exploring the differences in gut microbial composition between non-malnourished and malnourished patients with esophageal cancer. In this study, 16S rRNA high-throughput sequencing technology was used to analyze the composition and diversity of intestinal flora between non-malnourished and malnourished patients with esophageal cancer, and PICRUSt software was used to predict the differentially expressed functions and metabolic pathways, intending to provide novel therapeutic targets for early diagnosis and treatment of esophageal cancer. Our study divided esophageal cancer patients into non-malnutrition and malnutrition groups, conducted association analysis on bacterial communities, and observed significant changes. At the genus level, LEfSe analysis results showed that Dielma, Sellimonas, and Clostridioides were found to be significantly enriched among the non-malnutrition group, while Anaerococcus, Atopobium, Eubacterium_siraeum_group, and Lactobacillus were depleted. Notably, Dielma abundance seems positively associated with a better prognosis, aligned with Jeffrey Gordon's team findings (62). An increase in Clostridioides appears linked to better quality of life, but no significant discrepancy was found here due to random sampling errors. It's worth noting that Clostridioides incites infections, causing substantial health and financial burdens globally (63). Therefore, whether we can find an intermediate value of Clostridioides to explain this opposite result still needs further study.

Eubacterium_siraeum_group (64) is a core gut bacteria facilitating nutrient digestion and maintaining gut balance. A significant negative correlation with serum albumin in our study indicates that reduced levels might prevent protein loss, leading to elevated albumin levels bolstering immunity against diseases. In 2022, Jiangsu Province Hospital lodged a patent (65) focusing on Eubacterium_siraeum_groupi as a predictive tool for chemotherapy-induced cachexia, aiding in timely intervention and reducing illness severity. Research reveals (66) that Lactobacillus reuteri, a well-studied probiotic strain, produces antimicrobial molecules and modulates the gut microbiome. Possibly, it supports the host immune system by reducing proinflammatory cytokines while enhancing regulatory T cells. It fortifies the gut barrier and prevents inflammatory conditions such as IBD. However, this study shows that the more Lactobacillus, the higher the potential nutritional risk may be, but it may contribute to increased physical albumin and lean tissue. Further, larger sample sizes are needed to ascertain its effects on esophageal cancer patients.

This research also utilized the PICRUSt pipeline to foresee variations in functional and metabolic pathways between two patient groups based on their respective 16S KEGG functions. Our results highlight that the gut microbiota may affect disease progression by influencing sugar metabolism, DNA synthesis, or division pathway alterations. The study observed that the cell division topological specificity factor K06940 plays a pivotal role in triggering precise regulation of cell division. The reduced expression of its genus in malnourished patients could result in inaccurate regulation and impede normal growth and genetic information transfer. Uncharacterized proteins, pyrP, uraA, and uracil permease-K07461, may affect the synthesis, transport, and metabolism of uracil. In this study, the expression of the pathway in the malnutrition group was decreased, which may affect the normal transcription of DNA. Putative endonuclease can repair damaged DNA and process accounting fragments during DNA replication. The expression of putative endonuclease in the malnutrition group is reduced, which may affect the repair of DNA damage in patients with esophageal cancer. In summary, the alterations in underfed patients' related factors might limit effective DNA repair following damage and may affect the precise regulation of cell division and then participate in the occurrence and development of tumors, aligning with studies conducted by Wilson's team (67) and Till's team (68). In this study, lower uxaC and glucuronate isomerase [EC:5.3.1.12] levels in underfed individuals, both key components involved in glucose metabolism, uphold the importance of these processes for energy production and immune regulation. Related studies (69, 70) also demonstrated evidence of disrupted glucose metabolism amongst tumors, leading to tumor growth, diminished immunity, and severe energy deficiency, ultimately fostering cachexia.

Additionally, increased RP-L36, MRPL36, rpmJ, and large subunit ribosomal protein L36 levels suggested their potential role as markers or therapeutic targets due to their central roles in promoting cell viability and protein synthesis. Some studies (71) have shown that the large subunit protein L36 may become a potential target for drug therapy, especially in the development of anti-tumor drugs, and may become a potential tumor marker and therapeutic target. Further study of a larger sample size may be required to reduce biases and reconcile conflicting findings. In general, the relationship between gut microbiota and tumorigenesis is a complex and multidimensional problem. The mechanism of the relationship between gut microbiota, human health, and cancer is still in its early stages, mainly revealing correlation rather than causality.

Further in-depth research is needed to explore the mechanism. Several studies have demonstrated that manipulating gut microflora structure and metabolic product production can potentially prevent/treat some cancers. Our research shows that the intake of carbohydrates and fibers in patients with malnutrition or esophageal cancer is lower. Whether this affects the state of intestinal flora and thus affects the nutritional status of patients warrants further research.

This research has several limitations: First, it involves a single center with limited participant and subgroup numbers. Second, insufficient follow-up time precludes adequate analysis of OS data. Finally, the small sample size limits additional sub-group analyses due to high levels of confounders. Future studies necessitate larger-scale samples and prospective ones to verify these findings.

In conclusion, esophageal cancer patients face substantial nutritional risks and malnutrition rates due to their unique anatomical features. Nutritional status is correlated with carbohydrate intake, dietary fiber intake, protein levels, inflammatory levels, and lean body mass; this also affects the gut microbiota, influencing disease progression, and outcomes. These patients require early screening and intervention based on patient-specific indicators of nutrition; this might enhance their clinical outcomes. Given the interest in noninvasive and nonpharmacological interventions for such patients (72), dietary therapy and gut microbiome manipulation require further evaluation as lower-risk treatment options.

## Data availability statement

The data that support the findings of this study are available from the corresponding author upon reasonable request.

## Ethics statement

The study was approved by the Ethics Committee of the Chinese PLA General Hospital and registered in the Chinese Clinical Laboratory Registry under the registration number ChiCTR2100048141. The studies were conducted in accordance with the local legislation and institutional requirements. The participants provided their written informed consent to participate in this study. The requirement of ethical approval was waived by the study was approved by the Ethics Committee of the Chinese PLA General Hospital and registered in the Chinese Clinical Laboratory Registry under the registration number ChiCTR2100048141 for the studies involving animals because the study has a good specification. The studies were conducted in accordance with the local legislation and institutional requirements.

## Author contributions

LLi: Conceptualization, Data curation, Formal analysis, Investigation, Methodology, Project administration, Resources, Software, Supervision, Validation, Visualization, Writing – original draft, Writing – review & editing. ZX: Conceptualization, Data curation, Formal analysis, Investigation, Methodology, Project administration, Resources, Software, Supervision, Validation, Visualization, Writing – original draft, Writing – review & editing. HX: Project administration, Writing – original draft. LZ: Investigation, Writing – original draft. LLu: Investigation, Writing – original draft. LX: Project administration, Writing – original draft. LYe: Project administration, Writing – original draft. CJ: Project administration, Writing – original draft. ZK: Data curation, Writing – original draft. WH: Project administration, Writing – original draft. XJ: Data curation, Writing – original draft. CY: Project administration, Writing – original draft. CX: Data curation, Writing – original draft. LH: Data curation, Writing – original draft. YS: Data curation, Writing – original draft. LF: Supervision, Writing – review & editing. LYi: Supervision, Writing – review & editing.
